# Seropositivity of SARS-CoV-2 IgG Antibody among People in Dhaka City during the Prevaccination Period

**DOI:** 10.1155/2022/4451144

**Published:** 2022-01-27

**Authors:** Zabed Bin Ahmed, Mamudul Hasan Razu, Fatema Akter, Md. Raisul Islam Rabby, Pranab Karmaker, Mala Khan

**Affiliations:** Division of Life Science, Bangladesh Reference Institute for Chemical Measurements (BRiCM), Dr. Qudrat-e-Khuda Road, Dhanmondi, Dhaka 1205, Bangladesh

## Abstract

Severe acute respiratory syndrome coronavirus 2 (SARS-CoV-2) immunoglobulin G (IgG) detection can be an effective complementary tool to the reverse transcription–polymerase chain reaction (RT-PCR) test in estimating the true burden of coronavirus diseases 2019 (COVID-19) and can serve as baseline data, especially after the roll-out of vaccines against SARS-CoV-2. In this study, we aim to determine the seropositivity of SARS-CoV-2 IgG among people in Dhaka, Bangladesh. Volunteers, mostly asymptomatic people from Dhaka, were enrolled between October 2020 and February 2021. After obtaining participants' signed consents, blood samples were tested for SARS-CoV-2 IgG antibody, following the standard protocol of testing within 72 hours of collection. SARS-CoV-2 IgG was positive in 42% (101/239) of the cases. No difference was observed in terms of IgG positivity and IgG levels when stratified by age, gender, and blood group. However, RT-PCR-positive cases presented higher IgG levels compared to RT-PCR-negative/RT-PCR-not performed cases. SARS-CoV-2 IgG was found in 31% (32/102) and 28% (19/67) of RT-PCR-negative and RT-PCR-not performed cases, respectively. For RT-PCR-positive but SARS-CoV-2 IgG-negative cases (*n* = 13), the average time gap between the RT-PCR and SARS-CoV-2 IgG tests of six months indicates a gradual reduction of IgG. Eight cases for which samples were tested at two time points, three months apart, showed presented a decline in IgG levels with time (median IgG index of 2.55 in the first sample versus 1.22 in the second sample). Our findings reveal that several mild/asymptomatic cases that were RT-PCR-negative/not tested exist in the community, and IgG levels reduce in the human body over time.

## 1. Introduction

Coronavirus disease 2019 (COVID-19), first reported on December 30, 2019, in Wuhan Province of China, spreads rapidly worldwide, leading the World Health Organization (WHO) to declare it a pandemic on March 11, 2020 [1]. The first case of COVID-19 in Bangladesh was reported on March 8, 2020. Till April 26, 2021, 745,322 COVID-19 cases have been detected with 11,053 deaths. Thus, the overall prevalence of COVID-19 was 13.9%, with a case fatality rate of 1.48% [2]. Managing this pandemic entails halting the virus transmission or developing immunity among the masses against SARS-CoV-2 through vaccination programs. To reduce virus transmission, various steps such as hand sanitization, wearing of face masks, and social distancing have been enforced throughout the country. However, for a more sustainable and practical solution to address this pandemic, the government has introduced a vaccine in the country, which has resulted in several policy measures, such as contingency plans, vaccine introduction, and identification of priority groups for vaccination.

An understanding of the true extent of the disease in the country is critical to guide and implement these public health measures. Bangladesh, like most countries, opted for the reverse transcription–polymerase chain reaction- (RT-PCR-) based screening of only symptomatic patients. Despite this, the number of tests has been less than adequate. The detection of asymptomatic patients, their potential to spread the virus, and an inadequate number of tests indicate that the data generated from the country might be underestimated, and the true burden of the virus is much greater. According to the WHO, immunoglobulin G (IgG) and immunoglobulin M (IgM) surveillance against COVID-19 can help infer the extent of COVID-19 infection in the population [3].

Although RT-PCR is very sensitive and has been the mainstay for the detection of COVID-19 globally, the technique has its limitations from the policymaking perspective. The RT-PCR test is sensitive in terms of technical expertise, cost, transportation, and storage. The sensitivity of RT-PCR-based detection also depends on the timing of specimen collection [4]. As RT-PCR detects SARS-CoV-2 only in its active stages, this method is largely ineffective in detecting previous viral exposures [5].

Irrespective of the manifestation of symptoms, the human body produces antibodies in response to infections caused by various pathogens, including viruses. Given its relative simplicity compared to complex techniques such as RT-PCR, antigen/antibody test kits have been deployed, especially in epidemiological studies, as reliable instruments to measure the prevalence and patterns of infectious diseases [6]. Detection of antibodies, especially IgG—which is produced after two weeks of SARS-CoV-2 exposure and persists after the clearance of the infection—against SARS-CoV-2 can be an excellent indicator of previous, recovered, and undetected infections [7]. Therefore, it can effectively complement the RT-PCR test in understanding the true burden of COVID-19 in a country.

Despite its potential use in determining COVID-19 prevalence and addressing other scientific questions, data available on the seroprevalence of COVID-19 are mostly from developed countries, which differ from South Asian countries such as Bangladesh in terms of factors including infrastructure, culture, socioeconomic conditions, and care-seeking behaviors. With the vaccine against SARS-CoV-2 already introduced in the country, understanding the level of antibodies produced in response to the vaccine during the postvaccine period is critical. However, the absence of relevant data during the prevaccine period renders the understanding of the impact and dynamics of the vaccine difficult. Therefore, in this study, we report the baseline data on the seropositivity and level of IgG among people in Dhaka during the prevaccination period.

## 2. Methods

We enrolled volunteers, mostly from Dhaka, with or without symptoms of COVID-19 infection between October 2020 and February 2021. COVID-19 cases were defined as cases that were confirmed through the RT-PCR test. After obtaining signed consent, the volunteers were asked to complete a questionnaire to collect information. The questionnaire contains participant's demographic information (age, gender, area of living, blood group. etc.) and clinical information (presenting symptoms if any, history of comorbidity, etc.). Following the standard protocol, blood samples of 3 ml each were collected from the participants in a clot activator tube. The blood was centrifuged at 2,000 rpm for 5 min to separate the serum. The serum was separated and immediately stored at −20°C if not processed the same day. All blood serum samples were tested within 72 h of collection.

The sera were tested for SARS-CoV-2 IgG antibodies using the commercially available enzyme-linked immunoassay (ELISA) kit, ErbaLisa COVID-19 IgG. The ErbaLisa COVID-19 IgG kit is a European CE marked ELISA test kit that allows semiquantitative detection of SARS-CoV-2 IgG antibody in human serum samples with declared relatively high sensitivity and specificity of 98.3% and 98.1%, respectively [8]. All serum samples were tested according to the manufacturer's instructions. Briefly, 10 *μ*L of serum samples was diluted with 200 *μ*L commercially available sample diluent (1 : 20 dilution); 100 *μ*L of diluted samples was dispensed in specific microliter plates, and the subsequent steps were followed following the manufacturer's guidelines. The IgG antibody index was calculated to understand the level of IgG in the serum sample as per manufacturer's instruction: antibody index < 0.9 was considered as negative, and ≥0.9 was considered as positive.

We collected sequential samples for eight cases that presented positive results in the RT-PCR test at two time points: one between 14 and 30 days of the onset of infection and the other after 2 months of the first sample. These cases presented negative COVID-19 results in the RT-PCR tests between the periods of two sequential samples.

Multinomial logistic regression analysis was performed to compare SARS-CoV-2 IgG positivity among the different subgroups. IgG levels among different subgroups were compared using the Wilcoxon rank-sum test. All analyses were performed using the Stata software version 13.

## 3. Results

A total of 239 serum samples were collected between October 2020 and February 2021. The mean age of the patients was 42 ± 14.5 years (range: 5–80) with a male–female ratio of 4.5. The age of the majority of the participants (53%) was <40 years, with the highest share of participants (27.7%) being in the 20–30 years' age group. The lowest share of participants (14.3%) was in the age group of >60 years. The blood group was available for 206 cases, of which B positive was the most common (32.5%), followed by A positive (29.6%) and O positive (23.8%). The demographic characteristics of the participants are presented in Table 1.

**Figure 1:**
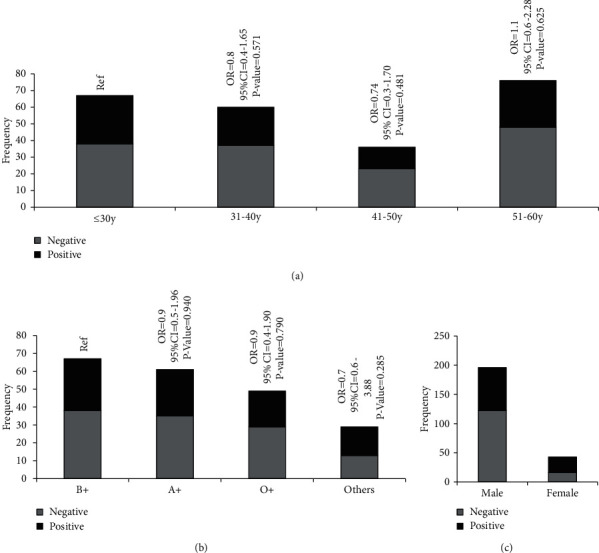
SARS-CoV-2 IgG seropositivity by (a) age group, (b) blood group, and (c) gender.

Overall, the seropositivity of the SARS-CoV-2 IgG antibody was found to be 42.2% (101/239). When stratified by age, gender, and blood group, no difference was observed in terms of IgG positivity (see Figure 1).

Cases for which both RT-PCR and SARS-CoV-2 IgG results were available numbered 227. Of the 102 cases that were reported as RT-PCR negative, 31% (32/102) were found to be positive for IgG. Alternatively, in 22.48% (13/58) of the RT-PCR-positive cases, no IgG antibody was detected. Out of these 13 cases, the duration between COVID-19 infection and SARS-CoV-2 IgG was available for 7 cases. The average duration between RT-PCR confirmed cases and the SARS-CoV-2 IgG test was 5 months (range: 3–6 months) for these 7 cases. SARS-CoV-2 IgG was found in 28% (19/67) of the patients who were never tested by RT-PCR (see Table 2).

**Figure 2:**
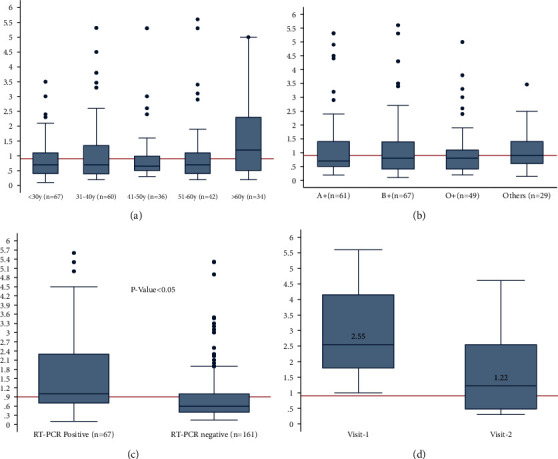
SARS-CoV-2 IgG index by (a) age group, (b) blood group, (c) RT-PCR results, and (d) at two time points.

The level of antibodies observed among the different age groups and blood groups did not differ significantly (Figures 2(a) and 2(b)). However, the antibody level of IgG in participants with RT-PCR-positive cases was found to be significantly higher: the median IgG index of RT-PCR-positive and-negative cases was 1.0 and 0.6, respectively (Figure 2(c)). Of the eight cases from which specimens were collected at two time points, all presented a gradual reduction in IgG levels over time. The median IgG index at the first and second visit was 2.55 and 1.22, respectively (Figure 2(d)). However, the degree of reduction in IgG levels varied among the individuals (data not shown).

## 4. Discussion

The seropositivity of IgG antibodies among the samples tested for our study, 6 months after the first detected case in the country, was found to be 42%. This is much higher than the community seroprevalence reported in other parts of the world: 2.8% in California, USA [9], 7.6% in Daegu, Korea [10], 5% in Spain [11], a comparable 22–33% in Iran [12], and 25.41% in Niger State, Nigeria [13]. This proportion indicates that the true burden of COVID-19 in Bangladesh might be much greater than that estimated by the RT-PCR-based detection method. This may be due to several mild/asymptomatic/recovered cases that were never tested using RT-PCR for various reasons or because of the ineffectiveness of RT-PCR in detecting previous infections. In terms of SARS-CoV-2 IgG seropositivity, no significant difference was found when stratified by the age and blood group. The male population was predominant in our study. Hence, we could not compare seropositivity and IgG index for SARS-CoV-2 in terms of gender.

Almost one-third (31.4%) of RT-PCR-negative cases were found positive for SARS-CoV-2 IgG, which further corroborates the assumption of several cases missed by RT-PCR-based techniques. Even in cases that were never tested by RT-PCR, approximately 28% were found to be positive for IgG. This indicates the underestimation of the burden of COVID-19 through RT-PCR, which is in concordance with the findings from other parts of the world [9]. For the RT-PCR-positive cases, we found 77.6% IgG positivity after 3–4 weeks of COVID-19 symptom onset. This is higher than the 45% IgG positivity among cases of RT-PCR-positive asymptomatic cases reported by Shirin et al. [14]. Interestingly, out of 58 RT-PCR-positive cases, we did not find detectable IgG antibodies in 13 cases. To investigate whether the long duration between the RT-PCR test and COVID-19 IgG may cause this, a further analysis among seven such cases (RT-PCR date was not available for the rest of the cases) demonstrated that the average duration between the RT-PCR confirmation and IgG test of these cases was 5 months (range: 3–6 months). By contrast, out of the remaining 45 RT-PCR-positive cases (which were IgG-positive), RT-PCR data were available for 24 cases. The average duration between the RT-PCR confirmation and IgG test of these 24 cases was 3.8 months (range: 1–7 months). This might indicate the possibility that the level of IgG reduces with time, and by the time these cases were tested, their IgG levels were below the detection level.

To test this possibility of reducing IgG levels even further with time, we randomly selected eight IgG-positive cases to examine the level of IgG at two time points: first, serum within 14–30 days of RT-PCR confirmation and second, serum 3 months after the first sample. We found that the median IgG index of these cases reduced with time: 2.55 in the first sample vs. 1.22 in the second sample. However, the reduction in IgG levels with time varied among individuals. This finding is similar to reports published in other parts of the world: Iyer et al. reported slow decay of SARS-CoV-2 IgG through 90 days after the onset of symptoms [7]. Long et al. also found a decline in SARS-CoV-2 IgG two weeks after discharge from the hospital [15]. Similar findings of a decrease in IgG levels with time were also reported by Ibarrondo et al. [16] and Campos et al. [17]. However, a later study reported that despite declining, IgG levels were detectable for more than six months.

The study has the following limitations: (1) the sample size was relatively small and participants enrolled voluntarily, which might not represent the true population of the country. Moreover, the study participants were heavily dominated by males. (2) For the longitudinal cohort to study IgG levels over time, we enrolled only eight cases and analyzed them for two time points. (3) We only checked SARS-CoV-2 IgG in this study. IgM and IgA and neutralizing antibody analysis as a whole are required for a comprehensive understanding of the dynamics of the SARS-CoV-2 antibody.

## 5. Conclusions

This is one of the first studies to shed light on SARS-CoV-2 IgG seropositivity in Bangladesh. Our results indicated that the seropositivity of IgG among the participants tested was 42%, and the IgG positivity did not differ in terms of age, gender, and blood group. We also revealed several mild/asymptomatic patients in the community, who either tested negative in the RT-PCR test or never tested for SARS-CoV-2. Our findings also demonstrated that with time, the level of IgG reduces within the human body. As Bangladesh recently introduced a vaccine against COVID-19, further comprehensive SARS-CoV-2 antibody analysis during the postvaccine period with large sample size is required to guide policy decisions.

## Figures and Tables

**Table 1:** Demographic characteristics of the participants.

Characteristics	Frequency	Percentage
Age		
≤30 y	67	28.0%
31–40 years	60	25.1%
41–50 years	36	15.1%
51–60 years	42	17.6%
>60 years	34	14.2%
Gender		
Male	196	82.0%
Female	43	18.0%
Blood group		
A+	61	29.6%
B+	67	32.5%
O+	49	23.8%
Others	29	14.1%
Unknown	33	13.8%
Symptoms		
Yes	18	7.5%
No	221	92.5%
RT-PCR		
Positive	67	28.0%
Negative	94	39.3%
Not performed	67	28.0%
Missing	11	4.6%

**Table 2:** Comparison of RT-PCR and SARS-CoV-2 IgG results (*n* = 227).

RT-PCR result	SARS-CoV-2 IgG result		Total
	Negative (%)	Positive (%)	
Negative	70 (68.6)	32 (31.4)	102
Positive	13 (22.4)	45 (77.6)	58
Not performed	48 (71.6)	19 (28.4)	67
Total	131	96	227

## Data Availability

The data used to support the findings of this study are included in this article.
